# Angiogenesis in pre-malignant conditions

**DOI:** 10.1038/sj.bjc.6604733

**Published:** 2008-10-21

**Authors:** S R Menakuru, N J Brown, C A Staton, M W R Reed

**Affiliations:** 1 Microcirculation Research Group, Academic Surgical Oncology Unit, School of Medicine and Biomedical Sciences, University of Sheffield, Sheffield S10 2JF, UK

**Keywords:** angiogenesis, microvessel density, pre-malignant, carcinogenesis

## Abstract

Evidence from human studies suggests that angiogenesis commences during the pre-malignant stages of cancer. Inhibiting angiogenesis may, therefore, be of potential value in preventing progression to invasive cancer. Understanding the mechanisms inducing angiogenesis in these lesions and identification of those important in human tumourigenesis are necessary to develop translational strategies that will help realise the goal of angioprevention.

Pre-malignant conditions are clinically recognisable lesions that are strongly associated with the development of malignant neoplasia. Pre-malignant lesions are defined by the epidemiological observation that patients with such lesions have an increased risk of cancer, and conversely, patients with cancer of a specific organ also display a high incidence of pre-malignant conditions (Peckham *et al*, 1995). It is therefore important to detect these precursor lesions and to stratify according to the risk of cancer development, thus allowing the identification of high-risk groups and for programmes of education, screening and prophylactic therapy to be developed (Kumar *et al*, 2005).

Pre-malignant lesions have been identified in almost all epithelial organs, and a distinct histological state termed dysplasia has been described (Table 1). This implies a disorderly but non-neoplastic proliferation, with loss of cellular uniformity and architectural organisation. Mild-to-moderate changes that do not involve the entire epithelium may be reversible with the removal of putative inciting causes. In contrast, carcinoma *in situ,* a lesion in which dysplastic changes are marked and involve the entire thickness of the epithelium, is considered to be a pre-invasive stage of cancer that possesses all the morphological features of cancer, except evidence of invasion beyond the basement membrane (Kumar *et al*, 2005).

Angiogenesis, the growth of new vessels from the existing vasculature, plays an essential role in tumour progression, providing nutrients and growth factors, in addition to aiding tumour cell dissemination (Hanahan and Folkman, 1996). Angiogenesis requires the release of growth factors mitogenic to endothelium, and the process is dependent on the net balance of angiogenic and antiangiogenic factors. The progression of an avascular tumour to the vascularised angiogenic phenotype is termed the ‘angiogenic switch’ (Folkman *et al*, 1989) and is likely to occur by multiple small steps resulting in a gradual increase in angiogenic potential, as the normal tissue acquires neoplastic features transforming initially to a pre-invasive cancer, with a subsequent progression to an invasive tumour in some cases. There is increasing evidence that the angiogenic process commences in the pre-malignant stages of most cancers based on experimental as well as clinical observations that challenge the previously held paradigm that malignant tumours induce angiogenesis at a volume of 1–2 mm^3^ (Gimbrone *et al*, 1972). There is also evidence that therapeutic interventions, which prevent angiogenesis, decrease the aggressiveness of malignancy; therefore, this approach may prevent or delay the progression of pre-malignant conditions to frank malignancy (Albini *et al*, 2007). In addition, evidence of increased angiogenesis in pre-malignant lesions may serve as a surrogate marker for tumour development, as the majority do not progress to malignant disease. Identification of the stage in the spectrum of disease, when the shift in the balance of factors influencing angiogenesis occurs, and the factors/mechanisms responsible for this shift is therefore important in developing strategies to prevent the progression of pre-malignant lesions to malignant tumours.

Although there are myriad pro- and antiangiogenic growth factors, based on knowledge gained from investigations of angiogenesis in malignant tumours, the following factors have been studied in human pre-malignant lesions. The most potent angiogenic growth factors belong to the vascular endothelial growth factor (VEGF) family. Vascular endothelial growth factor-A (commonly referred to as VEGF) is the most abundant family member acting as a specific mitogen for endothelial cells *in vitro* and as an angiogenic molecule *in vivo* (Ferrara *et al*, 2003). The primary regulator of VEGF secretion is the hypoxic microenvironment, which is mediated by the transcription factor hypoxia-inducible factor 1-*α* (HIF-1*α*). Under hypoxic conditions, HIF-1*α* accumulates in the cytoplasm and forms a heterodimer with HIF-1*β*. This, in turn, facilitates nuclear translocation of HIF-1*α*, where it initiates the transcription of genes involved in various cellular responses to hypoxia (Pugh and Ratcliffe, 2003). The actions of VEGF are mediated by a family of closely related tyrosine kinase receptors consisting of three members termed VEGFR-1, VEGFR-2 and VEGFR-3. Vascular endothelial growth factor receptor-2 is the principal pro-angiogenic receptor and mediates the majority of the downstream effects of VEGF-A (Ferrara *et al*, 2003). Thymidine phosphorylase (TP), an intracellular enzyme exhibiting angiogenic activity, stimulates endothelial cell proliferation and migration (Ishikawa *et al*, 1989). It also confers resistance to hypoxia-induced apoptosis. Matrix metalloproteinases (MMPs) facilitate angiogenesis by degrading the basement membrane and the ECM, which in addition to facilitating capillary formation also releases growth factors bound in the stroma (Folgueras *et al*, 2004). Discussion of all the growth factors and inhibitors involved in the phenomenon of angiogenesis is beyond the scope of this review. This review focuses on the current available evidence supporting the concept of angiogenesis in human pre-malignant lesions and the putative mechanisms regulating this phenomenon.

## Evidence for increased angiogenesis in human pre-malignant conditions

Experimental tumours less than 1 mm^3^ are in general avascular and grow slowly due to limitations imposed by the rate of diffusion of oxygen and nutrients. This was based on the observations that tumours grown in isolated avascular areas, such as the aqueous chamber of the eye (where blood vessels could not proliferate), expanded only to a size of 1 mm^3^, but after implantation into the iris, which is highly vascular, neovascularisation was induced and the tumour demonstrated rapid growth (Gimbrone *et al*, 1972). However, this paradigm was challenged by the RIP-TAG transgenic murine model of islet cell tumorigenesis (Hanahan, 1985). The mice express the large T antigen in their islet cells at birth and express the SV40 antigen under the control of the insulin gene promoter, resulting in a sequential development of tumours in the islets over a period of 12–14 weeks. Tumour development proceeds in stages; initially, approximately 50% of the islets become hyperproliferative with a subset (25%) subsequently acquiring the ability to induce angiogenesis, approximately 15–20% of which develop into benign and invasive tumours. It is evident from this model that angiogenesis commences well before the emergence of an invasive malignant phenotype (Folkman *et al*, 1989). Subsequently, considerable evidence has accumulated in favour of this concept from studies in human pre-malignant lesions, the majority of which have quantified angiogenesis using the surrogate measure of microvessel density (MVD). This is the mean microvessel count, obtained using a limited number of fields, subjectively selected and representing the most vascularised areas termed as ‘hot spots’ (Fox and Harris, 2004). The most commonly used antigens to characterise microvessels immunohistologically are Factor VIII, CD31 and CD34. It is now also possible to discriminate between newly formed immature and established mature vessels using CD105 (Fox and Harris, 2004). In addition, the expression of angiogenic growth factors by pre-malignant lesions is also considered to be an indicator of angiogenic activity. The following summarise the evidence for angiogenesis from individual human organs.

### Skin

Both epithelial and melanocytic pre-malignant lesions have been investigated for angiogenic activity. Immunohistochemical analysis of actinic keratoses and Bowen's disease from 35 individuals demonstrated both increased MVD and endothelial proliferation rate. Both parameters increase significantly at each disease stage, suggesting that angiogenic activity is increased early in the development of dermal squamous cell carcinomas (SCCs) (Nijsten *et al*, 2004). Pre-malignant lesions of the melanocyte lineage also demonstrate significantly increased MVD between benign naevi and dysplastic nodules (Barnhill *et al*, 1992).

### Respiratory tract

Angiogenesis has been identified in laryngeal leukoplakia, with MVD increasing significantly between normal epithelium and dysplasia and between dysplasia and carcinoma (Franchi *et al*, 2002). In bronchial pre-malignant lesions, a progressive increase in MVD from hyperplastic/metaplastic lesion to dysplasia or *in situ* carcinoma has been demonstrated. Vascular endothelial growth factor mRNA and protein expression parallel the increased MVD in these lesions, with VEGF expression predominantly in the bronchial epithelium (Merrick *et al*, 2005).

### Gastrointestinal tract

Studies of oral pre-malignant tissue demonstrated a significant increase in MVD with progression from normal epithelium to invasive cancer (Carlile *et al*, 2001). In a large series of Barrett's oesophagus and associated adenocarcinoma, angiogenesis was markedly increased in pre-malignant lesions, which correlated with VEGF expression. Vascular endothelial growth factor expression was elevated in epithelial cells with strong VEGFR-2 expression on associated vessels (Auvinen *et al*, 2002). Evidence of enhanced angiogenesis was also demonstrated in precursor lesions of oesophageal SCC accompanied by elevated VEGF and TP levels (Kitadai *et al*, 2004). Increased CD34 microvessel count and VEGF mRNA and protein expression was demonstrated in *Helicobacter pylori*-associated gastritis (Tuccillo *et al*, 2005). Vascular endothelial growth factor expression and inducible nitric oxide synthase were also elevated in chronic atrophic gastritis as well as in metaplastic and dysplastic areas before the onset of gastric cancer (Feng *et al*, 2002).

Investigations in our unit quantifying angiogenesis in the colorectal adenoma-carcinoma sequence using immunohistochemical staining for CD31 antigen suggest that a significant increase in MVD is induced with the onset of dysplasia, parallelled by a significant increase in VEGF expression at the same stage of progression (Staton *et al*, 2007). Microvessel density and VEGF expression also increase in a linear manner with the progression of transformation in anal intraepithelial neoplasia, the precursor of anal SCC (Mullerat *et al*, 2003). Angiogenesis, as defined by the extent of sinusoidal capillarisation detected by CD34 antibodies, increases with progression towards hepatocellular carcinoma (HCC) commencing at the low-grade dysplastic nodule stage with a concomitant increase in VEGF expression (Park *et al*, 2000).

### Genitourinary tract

Studies of vulvar intraepithelial neoplasia (VIN), the precursor of vulvar carcinoma, demonstrate a significant increase in MVD with increasing grade, which correlates with VEGF expression. Microvessel density alone is also a valuable tool in identifying potential progression to SCC in some cases of VIN III (Bamberger and Perrett, 2002). Several studies have concluded that there is a progressive increase in MVD from normal epithelium to SCC through cervical intraepithelial neoplasia, with some studies also demonstrating a significant correlation between MVD and VEGF expression (Dobbs *et al*, 1997; Tjalma *et al*, 1999). Thymidine phosphorylase expression also increases with lesion severity, but this does not correlate with MVD (Dobbs *et al*, 2000). In a large proportion of high-grade prostatic intraepithelial neoplasia, MVD is increased compared with normal tissue, which predicts concomitant prostatic carcinoma (Sinha *et al*, 2004).

### Breast

Although ductal carcinoma *in situ* (DCIS) has been studied extensively, increased vascularisation and VEGF expression has also been identified in both usual and atypical hyperplasia compared with normal ductal epithelium lesions thought to precede DCIS (Bos *et al*, 2001). Ductal carcinoma *in situ* lesions exhibit two patterns of angiogenesis, an increased stromal MVD and a periductal cuff of microvessels surrounding the basement membrane of the ducts. The tumour cells also expressed high levels of VEGF mRNA and VEGF protein, and there is increased VEGF receptor expression on the endothelial cells surrounding the tumours (Guidi *et al*, 1997).

Overall, evidence from human studies supports experimental observations from murine models, suggesting that initiation of angiogenesis occurs in the pre-malignant stages of cancer development. This is supported by the demonstration of the onset of angiogenesis, as determined by MVD, which precedes invasive cancer development in all the precursors of solid human cancers studied to date. There is also evidence supporting the premise that increased production of angiogenic growth factors by the tumour cells is responsible for the increased vascularisation of pre-malignant lesions. Although there is no consensus regarding increased MVD as a predictor of progression to malignancy, angiogenesis appears to be initiated during the pre-malignant stages and that progression to invasive cancer may therefore, at least in part, be facilitated by the increased supply of nutrients and oxygen provided by the increased vascular supply.

## Underlying mechanisms initiating angiogenesis in pre-invasive lesions

Substantial insights have been gained into the angiogenic mechanisms involved in malignant tumours. The subsequently developed antiangiogenic therapies demonstrated efficacy in the pre-clinical setting, and this has now been translated into clinical practice. Although it may be presumed that the same biochemical and physiological mechanisms are involved in the induction of angiogenesis in pre-malignant lesions, there is currently limited evidence to support this assumption. This could have been due to the paucity of experimental cell lines and animal models that are representatives of pre-malignant disease. Appropriate models of pre-malignant lesions are now being developed, which will assist in further understanding of the mechanisms involved (Heppner and Wolman, 1999; Tanaka *et al*, 2006). A combination of data from human tissue studies and investigations on experimental animal models has increased our understanding of some aspects of angiogenesis initiation in these lesions.

### Hypoxia-mediated angiogenesis

Overexpression of HIF-1*α* has been demonstrated in pre-malignant lesions of the breast, oesophagus and prostate (Bos *et al*, 2001; Griffiths *et al*, 2007), with HIF-1*α* levels correlating with VEGF expression and MVD in all the three organs. However, the lesions have no obvious areas of necrosis, suggesting that the upregulation of HIF-1*α* may be due to non-hypoxic mechanisms. This apparent paradox has been investigated in pre-malignant hepatic lesions (Tanaka *et al*, 2006). Oxygen tension was measured *in vivo* within murine pre-neoplastic hepatic lesions, which were shown to overexpress HIF-1*α* and its transcriptional targets such as VEGF and glut-1. Oxygen tension within these lesions was not different from that of normal liver tissue. However, the PI3K/Akt pathway, which can upregulate HIF-1*α* expression, was activated in these lesions and may be responsible for the non-hypoxic upregulation of HIF-1*α* expression (Tanaka *et al*, 2006). The activity of cellular kinases has also been found to be responsible for increased HIF-1*α* activity in hepatitis C virus (HCV)-infected hepatic cells. Hepatitis C virus, an RNA virus, promotes the development of chronic hepatitis and subsequent HCC (Nasimuzzaman *et al*, 2007). These observations suggest that genetic changes promoting carcinogenesis can result in an aberrant activation of hypoxic signalling in the cells of pre-malignant lesions under normoxic conditions.

### Inflammatory mediator-promoted angiogenesis

Inflammation has been known for many years to be an initiator and promoter of carcinogenesis and angiogenesis. There is now convincing evidence that inflammatory mediators are critical for the stromal changes supporting the development of cancer (Mantovani *et al*, 2008). Upregulation of cylooxygenase-2 (COX-2) has been widely reported in pre-malignant lesions and correlates with angiogenesis (Raspollini *et al*, 2007). Macrophage infiltration of pre-malignant lesions also correlates with neoplastic progression and angiogenesis (Mazibrada *et al*, 2008). These observations support the hypothesis that macrophages may be the source of several inflammatory mediators that promote angiogenesis including TNF-*α*, MMPs, COX-2 and chemokines. Our own *in vitro* investigations into the involvement of macrophages in tumour angiogenesis indicate that the infiltration of macrophages into breast cancer cell spheroids resulted in at least a three-fold upregulation in the release of VEGF when compared with spheroids composed only of tumour cells. The angiogenic response measured around the spheroids, 3 days after *in vivo* implantation into dorsal skinfold chambers, was significantly greater in the spheroids infiltrated with macrophages (Bingle *et al*, 2006). Evidence for similar role in pre-malignant lesions is provided by a transgenic mouse model of breast carcinogenesis in which inhibition of macrophage infiltration into tumours delayed the angiogenic switch whereas pre-mature induction of macrophage infiltration into pre-malignant lesions promoted an early onset of angiogenic switch (Lin *et al*, 2006). The transcription factor nuclear factor-kappa B (NF-*κ*B), a key controller of the inflammatory process, is activated by a large number of stimuli including microbial pathogens, tissue injury and necrosis among several others (Karin, 2006). Activated NF-*κ*B plays a critical role in inflammation-driven tumour progression as it influences multiple processes such as cell-cycle control, apoptosis, stromal protease production and angiogenesis in addition to the release of inflammatory mediators including interleukin-8 (IL-8), which promotes tubular morphogenesis of endothelial cells (Shono *et al*, 1996). It also acts as a chemoattractant, leading to the recruitment of monocytes, neutrophils and T lymphocytes to tumour sites, which release MMPs and chemokines stimulating angiogenesis. Increased activation of NF-*κ*B may be secondary to abnormal upstream pathways regulated by oncogenic tyrosine kinases such as the Ras/MEK/ERK pathway (Mantovani *et al*, 2008). In addition, there is evidence that HIF-1*α* and NF-*κ*B may act synergistically, further increasing angiogenesis (Scortegagna *et al*, 2008).

### Oncogene-mediated angiogenesis

Recently, evidence has accumulated that certain oncogenes can directly induce angiogenesis. These include the *RAS* and *MYC*, which are implicated in the development of multiple cancers. Ras proteins are a family of signal-transducing G proteins. Ras activates the MAP kinase pathway, which in turn targets nuclear transcription factors promoting mitogenesis (Shaw and Cantley, 2006). Nuclear factor-*κ*B is one of the proteins whose expression is increased by this process and can act as a promoter of angiogenesis. The *MYC* proto-oncogene is expressed in virtually all eukaryotic cells and belongs to a group of genes that are rapidly induced when quiescent cells receive a signal to divide. In a mouse transgenic model of Myc-dependent *β*-cell carcinogenesis, the onset of endothelial cell proliferation was noted to begin shortly after Myc-induced cell cycle entry of pancreatic *β* cells. Subsequent endothelial proliferation was not caused by local tissue hypoxia but through the release of pre-existing sequestered VEGF from the ECM by MMPs mediated by the production and release of the pro-inflammatory cytokine IL-1*β* (Shchors *et al*, 2006).

The tumour suppressor p53 acts as a suppressor of angiogenesis by regulating the production of thrombospondin-1, a potent angioinhibitor (Dameron *et al*, 1994). Decreased p53 activity correlates with increased expression of VEGF and angiogenesis in bronchial dysplasia and CIN (Fontanini *et al*, 1999; Lee *et al*, 2003). Phosphatase and Tensin homologue (*PTEN*) is a tumour suppressor gene that induces cell cycle arrest and apoptosis. Loss of PTEN protein occurs during pre-malignant stages of endometrial carcinoma (Mutter, 2002). PTEN also acts as an inhibitor of the PI3K/Akt pathway that regulates the synthesis of HIF-1*α*, and therefore loss of PTEN activity can lead to the promotion of angiogenesis (Lee *et al*, 2005).

This phenomenon has also been observed during human viral oncogenesis. Infection with the oncogenic human papilloma virus is an initiating factor in the carcinogenesis of uterine cervix. The high-risk oncoproteins, E6 and E7, are necessary for the immortalisation and transformation of cervical keratinocytes. The oncoprotein E6 binds to p53 protein, whereas E7 binds to the tumour suppressor RB (retinoblastoma gene product) and induces their degradation. In addition, the overexpression of these proteins in cervical cancer cells increases HIF-1*α* and VEGF expression, thus potentially promoting angiogenesis in cervical neoplasia (Tang *et al*, 2007). Hepatitis C virus infection is an important cause of HCC, and in HCV-infected hepatic cells, it has been shown that HIF-1*α* is stabilised under normoxic conditions, leading to an increased production of VEGF (Nasimuzzaman *et al*, 2007).

In summary, the angiogenic switch involves a reprogramming of the cellular transcription profile, with pro-angiogenic factors predominating over antiangiogenic factors, resulting in an angiogenic phenotype. The examples detailed reflect the way in which cellular events that promote cell cycling and proliferation during carcinogenesis are linked inextricably to stromal events that support the expansion of abnormal cells thus driving tumour progression.

## Therapeutic implications

Chemoprevention is the use of specific pharmacological or nutrient agents to prevent, reverse or inhibit the process of carcinogenesis. Recognition of the fact that many cancers have distinct pre-invasive phases, combined with the realisation that a number of these lesions appear to regress once the carcinogenic stimulus is removed, suggests that it may be possible to prevent the development of cancer. The discovery that angiogenesis and carcinogenesis are linked and that angiogenesis is dependent for growth has led to attempts to prevent carcinogenesis through combined angiopreventive strategies (Albini *et al*, 2007). Although traditional cancer chemoprevention relies on agents that prevent or delay the transformation of normal cells into cancer cells, angioprevention strategies target those cells in the microenvironment associated with angiogenesis. These two interventions targeting different cell types may act synergistically, resulting in a more effective prophylaxis than can be achieved with either individually. Advances in non-invasive imaging, facilitating the assessment of angiogenesis in tissues, may allow clinicians to monitor angiopreventive strategies (Barrett *et al*, 2007). Recent research has demonstrated that statins promote endothelial cell death and inhibit experimental angiogenesis induced by growth factors, providing ‘proof of principle’ for developing statin-based angiopreventive strategies (Boodhwani *et al*, 2006). Some flavonoids such as xanthohumol and deguelin have also been demonstrated to have angiopreventive properties under experimental conditions (Albini *et al*, 2007). These and the development of newer angiopreventive compounds in future may offer the ability to inhibit angiogenesis during pre-malignant stages and delay the onset of, or progression to, cancer.

## Figures and Tables

**Table 1:** Pre-malignant lesions and risk of their transformation into corresponding malignancies (compiled from Peckham *et al* (1995), except where indicated)

**Organ**	**Lesion**	**Cancer**	**Overall lifetime risk of transformation**
Skin	Actinic keratoses	SCC	10%
	Bowen's disease	SCC	Considered to be superficial SCC, 100 % risk of progression
	Dysplastic nodule	Malignant melanoma	Lifetime risk 100%
Larynx	Leukoplakia	SCC	5–10%
Bronchus	Bronchial dysplasia	SCC	10–40% (Merrick *et al*, 2005)
Oral cavity	Leukoplakia	SCC	5–10%
Oesophagus	Barrett's dysplasia	Adenocarcinoma	10%
	Epithelial dysplasia	SCC	90% of patients with tylosis (genetic disorder characterised by hyperkeratosis of palms and soles) progress to SCC
Stomach	Chronic gastritis	Adenocarcinoma	2–4%
Colon	Adenoma	Adenocarcinoma	10–15%
Anal canal	Anal intraepithelial neoplasia	SCC	5% (Mullerat *et al*, 2003)
Liver	Dysplastic nodule	Hepatocellular carcinoma	Unknown
Vulva	Vulvar intraepithelial neoplasia	SCC	5% of treated women progress to cancer, 90% untreated women progress (Raspollini *et al*, 2007)
Cervix	Cervical intraepithelial neoplasia	SCC	30–50% (Tjalma *et al*, 1999)
Prostate	Prostatic intraepithelial neoplasia	Adenocarcinoma	High-grade PIN is a marker for a high risk of concomitant or later (33–100%) carcinoma (Sinha *et al*, 2004)
Breast	Usual hyperplasia	Adenocarcinoma	Relative risk increased 1.5- to 2-fold (Arpino *et al*, 2005)
	Atypical hyperplasia	Adenocarcinoma	Relative risk increased four-fold (Arpino *et al*, 2005)
	Ductal carcinoma *in situ*	Adenocarcinoma	Relative risk increased 8- to 10-fold (Arpino *et al*, 2005)

SCC=squamous cell carcinoma.
